# Kansas

**Published:** 1896-08

**Authors:** 


*Kansas.* Texas fever, introduced through cattle from Indian Territory, has broken out to a certain extent near Independence. National and local officials are directing its control.

